# Tumor immune cell infiltration score based model predicts prognosis in multiple myeloma

**DOI:** 10.1038/s41598-022-21763-7

**Published:** 2022-10-12

**Authors:** Can Chen, Yiwei Li, Peiwen Miao, Ying Xu, Yaping Xie, Zhenzhen Chen, Shenxian Qian

**Affiliations:** 1 Department of Hematology, Affiliated Hangzhou First People’s Hospital, Zhejiang University School of Medicine, 216 Huansha Road, Hangzhou, 310006 Zhejiang China; 2 Department of Critical Care Medicine, Affiliated Hangzhou First People’s Hospital, Zhejiang University School of Medicine, 216 Huansha Road, Hangzhou, 310006 Zhejiang China; 3 Department of Hematology, Affiliated Hangzhou First People’s Hospital, Zhejiang Chinese Medical University, 216 Huansha Road, Hangzhou, 310006 Zhejiang China

**Keywords:** Cancer, Haematological cancer, Myeloma

## Abstract

The tumor microenvironment plays an important role in various processes, including tumorigenesis, cancer progression, and metastasis. Immune signatures have been identified and verified for use in diagnosis and prognosis prediction. We used single-sample Gene Set Enrichment Analysis to evaluate tumor immune cell infiltration score (TIICs) and verify their prognostic significance in both training and validation cohorts and using this information to build a prognostic model. A total of 1281 samples were obtained for further evaluation of the immune enrichment scores of 28 immune cells, showing that Th17 cell contributed most significantly to survival. Using the median TIICs as a cutoff to divide the samples into two groups, we found that the high-TIICs group was associated with favorable outcomes in both the training and validation sets. We then constructed a prognostic model to predict the 6, 8, and 10-year survival outcomes. Further analysis showed that immune score and tumor purity were higher in the high-TIICs group, while the matrix score was lower in this group. Forty-two differentially expressed genes were identified between the two groups. This new prognostic model based on immune cell infiltration indicates the potential for TIICs in predicting prognosis and as targets for treatment.

## Introduction

Multiple myeloma (MM) is characterized by the presence of clonal malignant plasma cells in the bone marrow. Symptoms include bone pain, anemia, recurrent infections, hypercalcemia, and renal failure. It is the second most common hematological cancer^1,2^. Progression of MM results from dysfunctional immune activity, including abnormal immune infiltration^3^. The tumor microenvironment contains a variety of cell types and molecules, including immune, mesenchymal, and endothelial cells, as well as extracellular matrix components and inflammatory factors. The proportion of infiltrating immune cells is closely associated with both cancer development and spread, and the use of this parameter may provide a new perspective for cancer research. Single-sample Gene Set Enrichment Analysis (ssGSEA) is a new method for analyzing the degree of immune invasion that was first published in Nature in 2009^4^ and has proved useful in calculating the scores of 28 immune infiltrating cells for 20 cancers, including breast cancer and colorectal cancer^5,6^. As it is well-known that immune function plays a major role in MM, a detailed analysis of the relationship between immune system genes and cancer outcomes may suggest new directions for both outcome prediction and treatment in MM.

To date, there has been no investigation of the role of the tumor immune cell infiltration score (TIICs) in the prognosis of MM. A straightforward and robust model based on immune function in MM would thus be extremely useful. Here, we used ssGSEA to determine the TIICs and assigned MM patients to two groups based on high and low TIICs scores. The value of the TIICs in predicting patient outcomes was verified using the training and validation cohorts. The findings indicated that immune-related gene signatures are valuable in predicting MM outcomes and may, in addition, also explain the underlying pathology of the disease.

## Materials and methods

### Data download, integration, and preprocessing

The chip and clinical data for four data sets, GSE136324, GSE4581, GSE136337 were downloaded from the GEO database (https://www.ncbi.nlm.nih.gov/geo/). TCGA:MMRF-COMMPASS was downloaded from the TCGA databas (https://xena.ucsc.edu/). The first two were used as the training set, and GSE136337 and TCGA:MMRF-COMMPASS as the validation set. The chip probe was first transformed into the gene name and the empty probe was removed. Then, multiple probes corresponding to the same gene were used to determine the median, which was used as the gene expression value. The batch effect of the GSE 136324 and GSE 4581 data sets was removed using the combat function in R's “SVA” package. A total of 4677 immune-related genes from 28 immune cells were obtained from the InnateDB database (https://www.innatedb.com/).

### Using ssGSEA to evaluate the proportion of infiltrating cells

ssGSEA is a new method for evaluating the degree of tumor immune infiltration^7^. ssGSEA has been used to calculate the scores of 28 immune infiltrating cells for 20 cancers. The method has also been used to evaluate infiltration in non-small cell lung cancer. Here, we used ssGSEA to calculate the TIICs of the three data sets, as well as determining the pairwise correlations between immune cells^8^. Cells showing significant correlations (P < 0.0001) were selected, unsupervised clustering of all immune cells was performed and the immune cell pairs displayed as a network diagram. Univariate Cox regression was used to identify the immune cell types related to outcomes in terms of prognosis and survival (P < 0.05).

### Construction of the model for immune infiltration as a prognostic indicator

Immune cells that showed significant correlations with prognosis were identified by univariate Cox regression. The TIICs was calculated according to the formula TIICs $=\sum_{i=1}^{n}\frac{1-HRi}{SE(HRi)}\times GSVA \left(cell\right)$, where HR is hazard ratio of immune cells, SE is the standard error of HR, and GSVA (Cell) is the enrichment score of immune infiltrating cells. The median TIICs was calculated and used as a cutoff to assign samples to high and low-score groups. Differences between the high- and low-TIICs groups were assessed using Kaplan–Meier survival curves.

### Validation of the external data set of the model

The validation cohort included 28 immune cell types with TIICs calculated by ssGSEA. The TIICs scores for prognosis were calculated in the same way. Kaplan–Meier curves were used to calculate the relationships between the TIICs and outcomes, and to examine whether there was a significant difference in prognosis between the high- and low-TIICs groups.

### Comparison of single immune cells with the model, and analysis of differences in risk between the high- and low-infiltration groups

The area under curve (AUC) values predicted by the TIICs and the seven immune cell types correlated with outcomes were determined in relation to clinical outcomes to assess their prognostic efficacy. Differences between the high and low-score TIICs groups were evaluated by the Wilcox test (P < 0.05).

### Multivariate Cox regression of the training and validation sets

Multivariate regression analysis was conducted based on the clinical information for the samples. However, there was insufficient clinical information in samples of the training cohort and the validation cohort (the first and third data sets included only information on survival but not on age or sex, amongst other parameters). Because of this, the training and validation cohorts were analyzed by univariate regression only. Nomograms were constructed based on the multivariate analysis, using various biological and clinical parameters, with the “RMS” package in R. However, only the second data set could be used for nomogram construction because of the lack of sufficient clinical information in the other two data sets.

### Differentially expressed genes and feature comparison between the two groups

Data on gene expression, patient survival, and TIICs were compared between the high and low-TIICs groups. The “limma” package in R was then used to identify differentially expressed genes (DEGs) using the thresholds |logFC|> 0.5 and P < 0.05, and the “gseGO” function was used to screen related pathways.

The “estimate” package in R was used to determine the immune score, matrix score, and tumor purity of the samples. Using this information, together with the TIICs grouping, differences between the groups in terms of these parameters were assessed by the Wilcox test (P < 0.05).

### Ethics declarations

Our study is based on open source data. The Hangzhou First People’s Hospital Research Ethics Committee has confirmed that no ethical approval is required.

## Result

### Data preprocessing and calculation of TIICs

**Figure 1:**
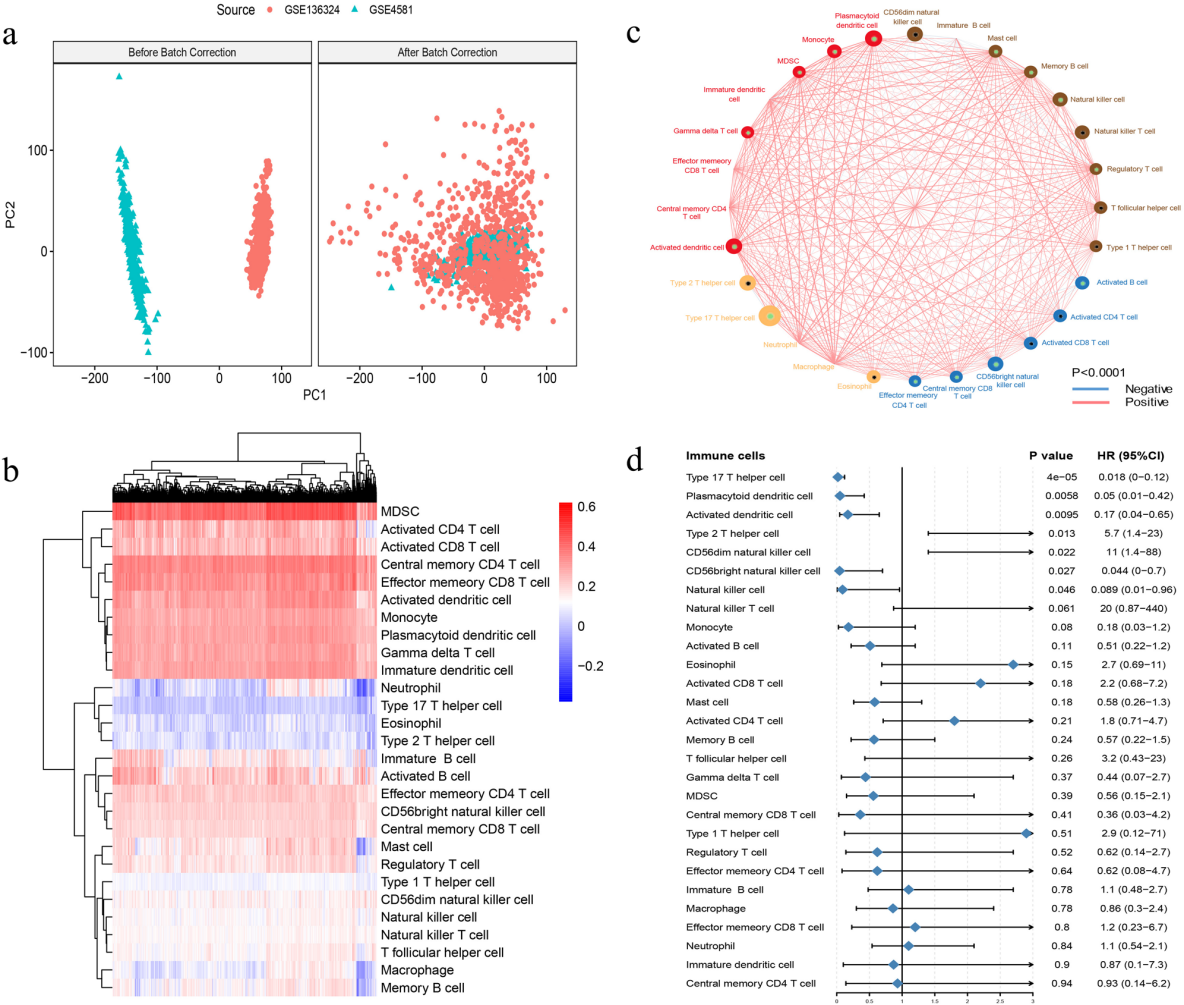
(**a**) There is no difference between GSE4581 and GSE136324 after the remove of cross-platform batch effects. (**b**) The 28 types of immune cells of enrich and infiltrate in different samples. (**c**) Seven cell types are identified that significantly influenced prognosis by using univariate Cox regression. (**d**) And unsupervised clustering classify four cell-type groups.

The GSE4581 and GSE136324 data sets were obtained from the GEO database and comprised the training set. After removal of cross-platform batch effects, no difference was observed between the two data sets (Fig. 1a). We used the ssGSEA algorithm to calculate the TIICs of the samples in the training set, identifying 1281 samples to be used for further analysis of the immune enrichment and infiltration of 28 types of immune cells. The distribution between the immune cell types in the different samples is shown in Fig. 1b.

Univariate Cox regression of the link between immune cells and prognosis indicated seven cell types that significantly influenced prognosis (Fig. 1c). Among all the 28 immune cell types, seven cells were found to correlated with prognosis, of these, type 17 Th cells contributed the most to survival. We further use unsupervised clustering to classify four cell-type groups (Fig. 1d). Pearson correlation coefficients between immune cell pairs were also calculated. The immune cell types significantly related to prognosis were selected based on the regression analysis, and the TIICs was determined based on the immune infiltration score.

### Validation of the prognostic value of TIICs

**Figure 2:**
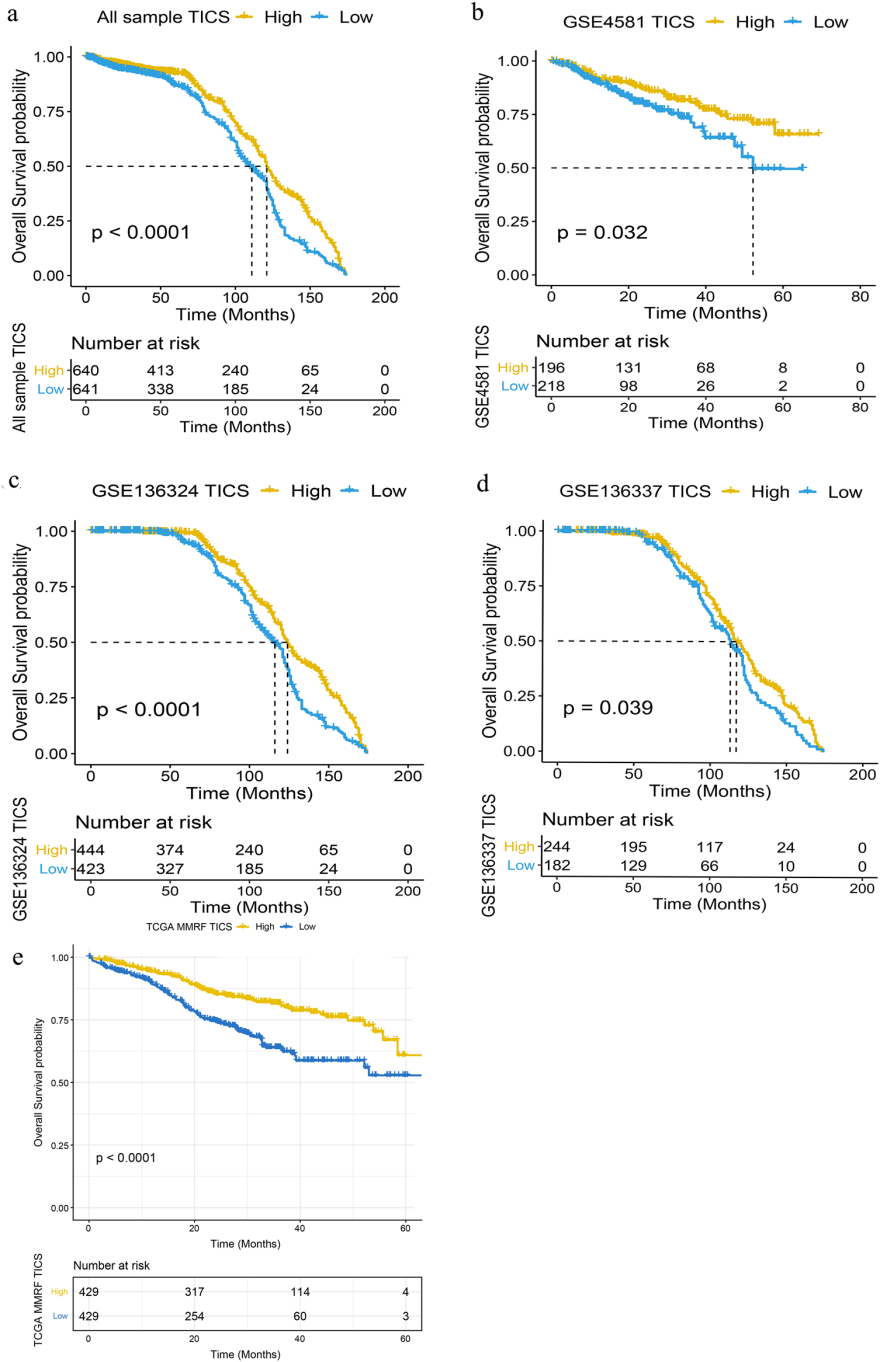
Kaplan–Meier analysis show that the high-TIICs groups had significantly better prognoses in the internal validation groups ((**a**), P < 0.0001), the GSE4581 ((**b**), P = 0.032) and the GSE136324 data sets ((**c**), P = 0.032). A further 426 samples confirmed that the high-TIICs group had longer OS ((**d**), P = 0.039). Validation datasets TCGA-MMRF confirmed the prognostic significance of TIICs for OS ((**e**), P < 0.0001).

Both internal and external validations were performed, and Kaplan–Meier analysis was performed for each data set with the median TIICs used as cutoff. We first analyzed the prognostic value of TIICs in the internal validation groups, finding that the high-TIICs groups had significantly better prognoses (Fig. 2a , P < 0.0001). Similar results were observed in the GSE4581 and GSE136324 data sets (Fig. 2b,c, P = 0.032, P < 0.0001 respectively). We included a further 426 samples and TCGA dataset (MMRF-COMMPASS) for independent external validation, using Kaplan–Meier analysis as above. This confirmed that the high-TIICs group had longer OS (Fig. 2d, P = 0.039, Fig. 2e, P < 0.0001). In order to confirm the prognostic significance of TIICs, we performed gene microarray in eight MM patients from our institution. The TIICs were calculated and found the high group has deeper response than low group. Limited to the number of samples and short time of follow up, we can not identify the difference of OS between the two groups. While patients in high group are all alive at last follow up compared to half of death in low group.

**Figure 3:**
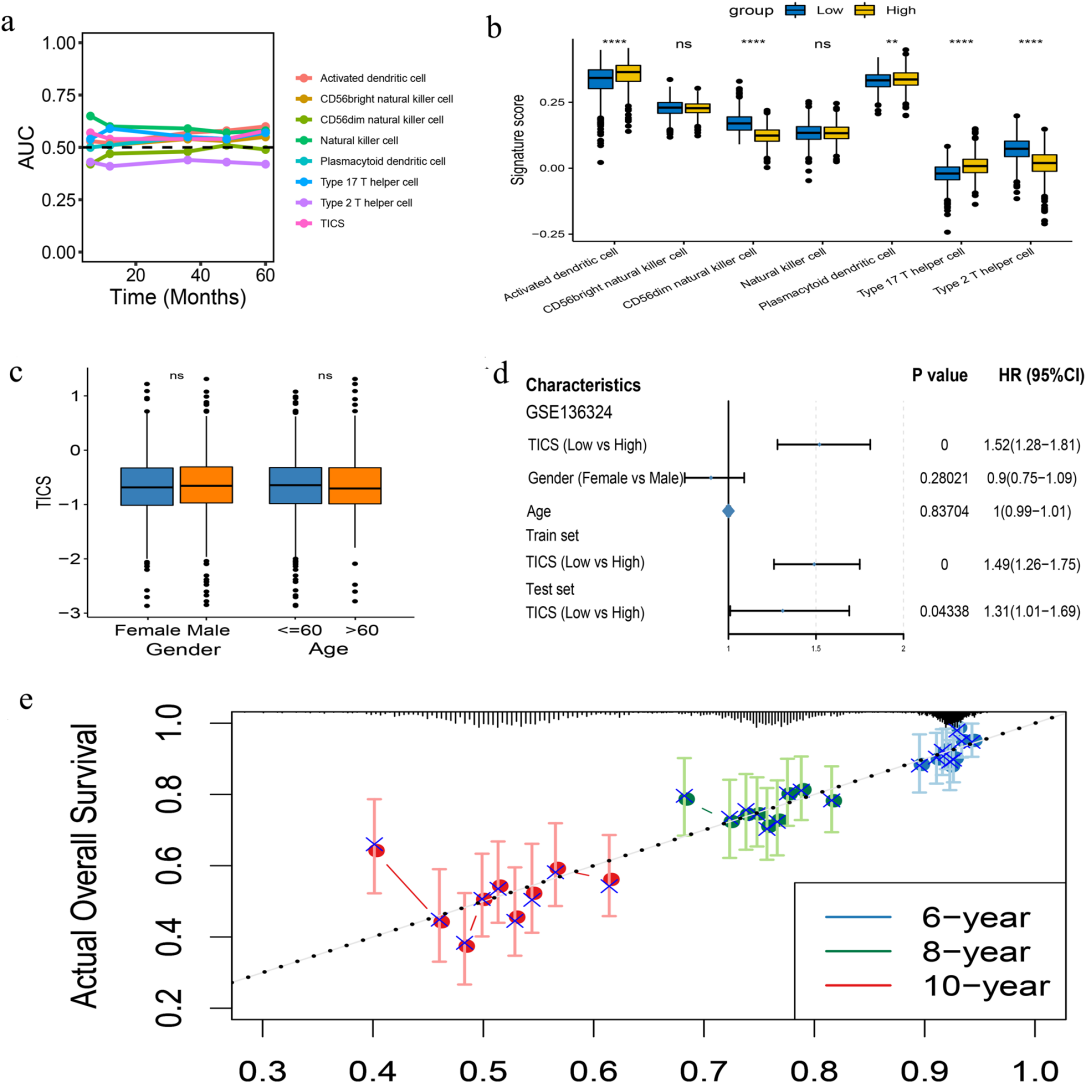
(**a**) Apart from natural killer cells, TIICs shows the high prognostic value in relation to the other cells. (**b**) Among seven cell types, five of them show significant different infiltration between the two TIICs groups, except for NK and CD56 bright NK cells. (**c,d**) There is no difference in age and gender in GSE136324 between the high and low-TIICs groups. (**d**) The remaining two data sets were analyzed by univariate regression which showed that TIICs was significantly correlated with prognosis. (**e**) A prognosis model including TIICs, age, and sex showed can ideally predict prognosis of 6-year, 8-year, and 10-year survival.

We next used AUC to compare the prognostic values of TIICs and the seven types of infiltrating cells. Apart from natural killer (NK) cells, which were significantly associated with OS, TIICs showed high prognostic value in relation to the other cells (Fig. 3a). The differences in the degree of infiltration by these seven types showed significant differences between the two TIICs groups for all cells except for NK and CD56bright NK cells (Fig. 3b).

### Multivariate Cox regression analysis of training set and validation set

Only the GSE136324 data set included detailed clinical data. The Wilcox test was used to determine the presence of significant differences in the clinical characteristics included in GSE136324 between the high and low-TIICs groups (Fig. 3c). The two groups did not differ in terms of age or sex (P > 0.05). Thus, the remaining two data sets were analyzed by univariate regression which showed that TIICs was significantly correlated with prognosis (Fig. 3d). We then constructed a prognosis model including TIICs, age, and sex using multivariate Cox regression. This model could predict the 6-year, 8-year, and 10-year survival (Fig. 3e).

### Differences in other factors between high and low-TIICs groups

**Figure 4:**
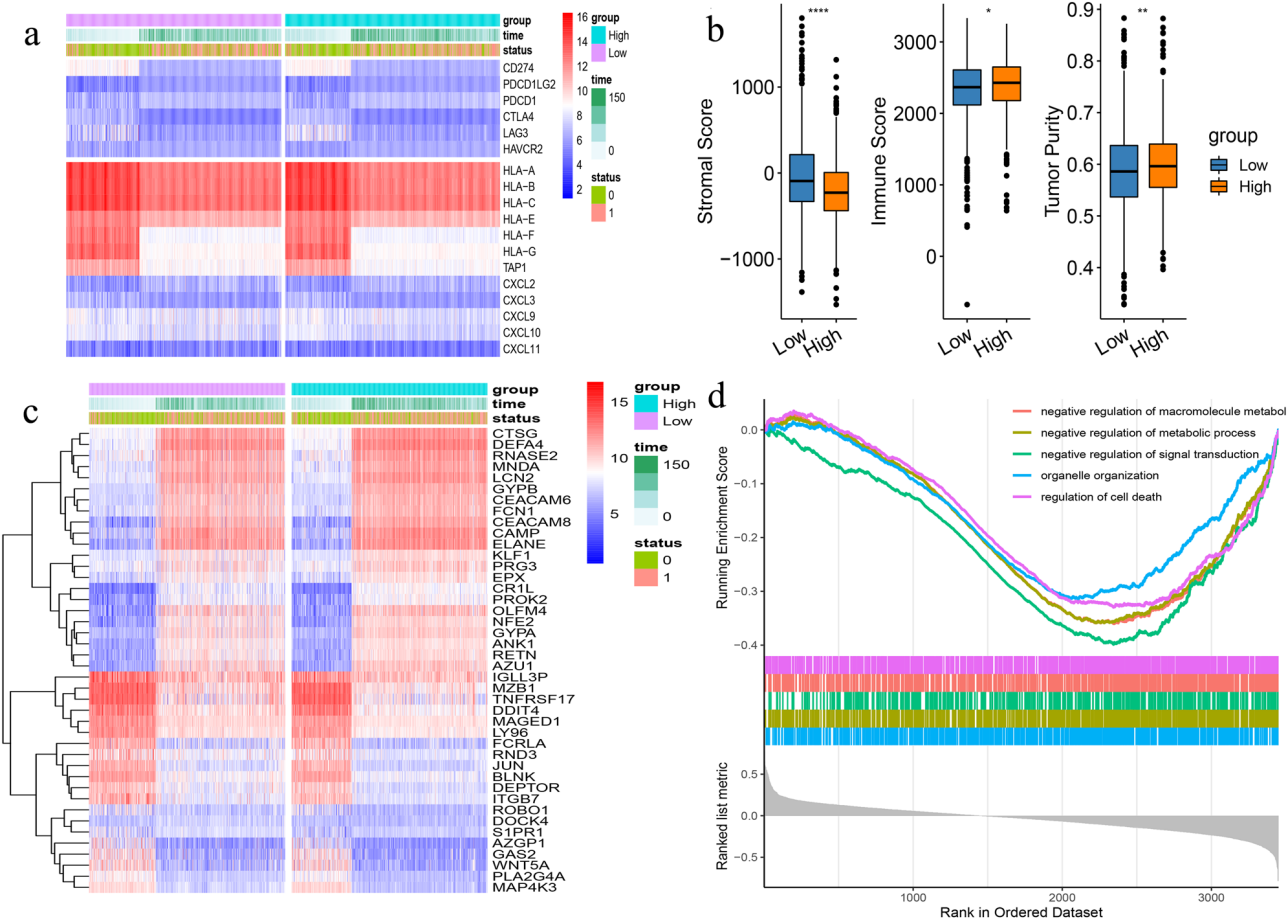
(**a**) There is no differences of the expression of immune checkpoint and chemokine genes between the two TIICs groups. (**b**) Both tumor purity and the immune score were higher in the high-TIICs group, while the matrix score was lower compared with the low-TIICs group. (**c**) 42 genes expressed differentially between the two groups were identified. (**d**) These genes were enriched in the categories “negative regulation of macro cellular metabolic”, “negative regulation of metabolic process”, and “negative regulation of signal transduction pathways”.

To examine possible further differences between the two TIICs groups, we investigate the expression of immune checkpoint and chemokine genes, observing no difference between the groups (Fig. 4a). However, the matrix score, immune score, and tumor purity were significantly related to clinical parameters, gene expression, and biological features, shown by the “estimate” package in R. Both tumor purity and the immune score were higher in the high-TIICs group, while the matrix score was lower compared with the low-TIICs group (Fig. 4b).

Forty-two genes expressed differentially between the two groups were identified by InnateDB (Fig. 4c). Of these 42 genes, DOCK4 and S1PR1 showed the greatest differences in expression. GO analysis using “gseGO” showed that genes with reduced expression were enriched in the categories “negative regulation of macro cellular metabolic”, “negative regulation of metabolic process”, and “negative regulation of signal transduction pathways” (Fig. 4d).

## Discussion

MM is characterized by the abnormal clonal expansion of antibody-secreting plasma cells in the bone marrow^9^. The incidence is approximately 3–4% of the over-50 population^10^. MM is twice as prevalent in the black population, and is also more common among males^11,12^. No specific genetic mutations have been identified as responsible for the disease^13^. However, recent evidence has suggested a link between MM and infiltrating immune cells^14^. Encourage results were reported by Danziger et al. They characterized the whole bone marrow microenvironment of MM and focused on prognostic significance of granulocyte signatures. They highlighted the importance of myeloid cells including granulocytes in tumor behavior and treatment response^15^. We have, therefore, using datasets part from this largest study to investigate the relationship between immune infiltration and the cancer, to determine both the pathogenetic mechanism and to suggest new directions for MM treatment.

ssGSEA is a recognized as one of the most reliable methods for investigating gene enrichment in individual samples^4^. In this study, the ssGSEA algorithm was used to calculate the TIICs in the training set samples (GSE136324, GSE4581). This identified 28 immune cell types from 1281 samples which were then divided into four enrichment-based clusters by application of the unsupervised hierarchical clustering algorithm. We found that type Th 17 cells contributed the most to survival outcomes, and seven survival-related immune cell types were identified. We then calculated the TIICs according to the immune cell infiltration scores, dividing the training cohort into two groups based on the median TIICs score cutoff. We found that the patient survival time differed significantly between the high and low-TIICs groups, verifying this finding in the validation cohort. Kaplan–Meier analysis showed that the high-TIICs group, in both the separate and combined data sets, had a significantly better prognosis. The nomogram was able to predict the 6-, 8-, and 10-year survival for MM. Comparing the differences between the two groups, we observed that they did not differ in terms of age and sex, nor of immune checkpoint and chemokine expression, while differences in immune score, stromal score, and tumor purity were noted. Forty-two DEGs were identified and investigated for pathway enrichment, showing negative regulation of the “macromolecule metabolic”, “negative regulation of metabolic process”, and “negative regulation of signal transduction” pathways.

Immune infiltration varies among different cancers and, even in the same tumor type, differences may be seen. The importance of this has been emphasized in MM pathogenesis. Immune-related signatures have been determined in different malignancies. Guo et al. has recently demonstrated the efficacy of a random-forest model based on immune infiltration in the differential diagnosis of several bone-marrow-related cancers, including MM^16^. As yet, there is no model based on immune cell signatures. Such a model would assist both in predicting outcomes and guiding treatment options and development.

There are four main subsets of naive CD4+ T cells, including Th1 and -2 cells, T regulatory (Treg), and Th17 cells^17^. Th17 cells have been observed to protect against infection and are involved in both inflammation and autoimmunity^18,19^. Th17 cells can also stimulate the secretion of IL-1α, IL-13, IL-17, and IL-23, as well as promoting myeloma cell growth, colony formation, and growth of xenografts in mouse models of MM^20^. IL-17 appears to be critical to these processes, and is secreted from MM cells^20^. Elevated Th17 cell numbers are seen in the peripheral blood of newly diagnosed MM patients as well as in other cancers^21^. The level of Th17 cells is differ from disease status, it will increase in PR and decreased in newly diagnosed patients in CR but further increased again when disease progressed^21^. In addition, the balance between Th17 and Tregs appears to be important in modulating the immune response; higher Th17 and lower Treg numbers were observed in long-term MM survivors^22^. Such as in this study, Th17 cells might affect the prognosis of MM via a complicated immune cells interaction. This indicated that Th17 cells may thus play an important role, not only in MM pathology but also in predicting the outcome of the disease.

NK cells have a variety of anti-tumor and immunomodulatory actions. These are usually classified as CD3−CD56+ cells in humans. Many studies have shown that NK cells are increased in the bone marrow of MM patients^23^. However, the expansion in the NK population is not associated with NK cell activation. Reduced NK activity has been linked to various parameters associated with tumor aggression, including advanced tumor stage and elevated levels of lactate dehydrogenase in MM^24^. In particular, CD56(bright)CD16(−/dim) NK cells, appear to be linked to cancer progression. In this study, it was found that NK cells were better able to predict prognosis than TIICs. Indeed, the activation of NK cells is essential to the determination of clinical stage and risk stratification, as well as drug response in MM patients. Several studies reported that, at least in part, the therapeutic efficacy of novel anti-MM drugs is associated with NK cell activation^25^. It is thus reasonable to develop cell immune therapy focusing on NK cells such as BCMA CAR NK.

Th2 cell differentiation from naive CD4 T cells has been linked to IL-4R induction of JAK1/3^26^. Th2 cells have also been related to MM prognosis^17,26,27^. Dendritic cells (DC) are differentiated from a lympho-myeloid hematopoietic pathway and are induced to specialize further by IRF8 and IRF4^28^. Plasmacytoid DCs secrete large amounts of type I interferon which not only modulates the immune response but also plays a part in tumorigenesis^29^. DC levels in MM are still controversial, due to differences in DC identification and quantification methods^30^. It is possible that DCs directly influence MM pathology, suggesting they may be potential therapeutic targets^25^.

We investigated multiple factors that may contribute to the differences between the two TIICs groups. Tumors contain not only malignant cells, but also non-malignant cells such as stromal, mesenchymal, and immune cells. These cells influence both the growth and progression of the tumor. The purity of tumors is known to be related to clinical parameters, tumor functioning, and gene expression, and should be considered as a confounding factor during analysis. Low tumor purity has been related to poor prognosis in colon cancer, glioma, and gastric cancer^31–33^. Similarly, in the current study, the low-TIICs group had lower levels of tumor purity which was linked to reduced survival. Meanwhile, we identified 42 DEGs included DEPTOR, EPX, and ROBO1. It has been shown that DEPTOR is necessary for myeloma differentiation and that higher levels are linked to improved outcomes^34^. ROBO1 functions as a proto-oncogene in MM promoting migration and proliferation through interactions with the bone marrow^35^. These genes enriched in negative regulation of macro cellular metabolic, metabolic process and signal transduction pathways that may affect the prognosis of MM.

There are some limitations of this study. Firstly, due to the limitations in clinical information, we were not able to compare the prognostic model with multiple clinical characteristics. We were also not able to identify clinical differences, such as stage and risk stratification between the groups. Moreover, survival was only measured over one to three years, so the prediction model of the nomogram could only be constructed for 4 to 5 years.

In conclusion, patients were classified into two groups based on TIICs, and the groups were shown to differ significantly in terms of immune risk, survival, and tumor environments. Thus, the identified immune cell risk signature may represent the MM tumor environment, allowing the prediction of patient prognosis and suggesting new directions for MM treatment.

## Data Availability

The datasets used and analyzed during the current study are available from the corresponding author upon request.
